# Evaluating the Effect of Daily Diary Instructional Phrases on Respondents’ Recall Time Frames: Survey Experiment

**DOI:** 10.2196/16105

**Published:** 2020-02-21

**Authors:** Arthur A Stone, Cheng K Fred Wen, Stefan Schneider, Doerte U Junghaenel

**Affiliations:** 1 Center for Self-Report Science and Center for Economic and Social Research University of Southern California Los Angeles, CA United States; 2 Department of Psychology University of Southern California Los Angeles, CA United States

**Keywords:** end-of-day dairy, daily diary study, recall time frame

## Abstract

**Background:**

Daily diaries are extensively used for examining participants’ daily experience in behavioral and medical science. However, little attention is paid to whether participants recall their experiences within the time frames prescribed by the task.

**Objective:**

This study aimed to describe survey respondents’ self-reported recall time frames and to evaluate the impact of different daily diary items on respondents’ reported affective states.

**Methods:**

In this study, 577 participants completed a mood survey with one of the following 4 time frame instructions: (1) *today*, (2) *since waking up today*, (3) *during the last 24 hours*, or (4) *in the last day*. They were also asked to indicate the periods they considered when answering these items and to recall the instructional phrases associated with the items.

**Results:**

Almost all participants in the *today* (141/146, 96.6%) and *since waking up today* (136/145, 93.8%) conditions reported using periods consistent with our expectations, whereas a lower proportion was observed in the *during the last 24 hours* (100/145, 69.0%) condition. A diverse range of responses was observed in the in the last day condition. Furthermore, the instructions influenced the levels of some self-reported affects, although exploratory analyses were not able to identify the mechanism underlying this finding.

**Conclusions:**

Overall, these results indicate that *today* and *since waking up today* are the most effective instructional phrases for inquiring about daily experience and that investigators should use caution when using the other 2 instructional phrases.

## Introduction

The daily diary method is an ambulatory assessment approach used by studies interested in assessing individuals’ experience over time in their natural environment. Daily diary studies involve study participants answering questions about their experiences (eg, mood, social interactions, location, and symptoms) on the internet via platforms such as Qualtrics or Assessment Center or via smartphones or other electronic devices in natural settings over many days. The resulting repeated data provide researchers with day-level data across the assessment period, which affords the opportunity to examine within-person processes that cross-sectional data cannot offer [1]. In addition, by inquiring about the day that has just passed at the end of the day in natural settings, end-of-day (EOD) daily diary methods provide data with improved ecological validity and reduced recall bias, compared with other study designs with longer recall periods [2]. A growing number of studies have taken advantage of the methodological strengths offered by daily diary methods, including clinical trials that evaluated treatment effects on patient-reported outcomes [3-5] and observational studies that tracked patient symptoms or healthy individuals’ daily experiences [6-11].

An important assumption of the EOD diary method is that it provides data reflecting the participants’ experience for the day. Diary investigators have used different instructional phrases to define the period of time that participants should consider when making their ratings. The 4 commonly used instructional phrases are *today* [8,12-14], *since waking up today* [15,16], *during the last 24 hours* [3,17], or *in the last day* [18,19]. At face value, some of the instructional phrases seem clear as to the time frame they intend to target, whereas others are, we believe, open to interpretation. For example, the phrase *today* and *since waking up today* clearly specify that the time frames of interest are within the current day. The phrase *during the last 24 hours* also appears to have a clear literal meaning—the investigator is asking about the previous 24 hours from the start of the questionnaire. In this case, if the diary were completed at 6 PM, then the recall period that participants should use would include the period beginning at 6 PM on the previous day. However, the phrase *in the last day* is less straightforward to interpret. Although investigators may intend for the phrase to inquire information about the day that has just passed (ie, today) [18,19], some may intend for the phrase to include parts of yesterday (ie, the previous night). However, whether EOD diary study participants assess their experiences with the prescribed time frames has not been examined.

There are many ways that study participants could interpret the recall instructions of EOD diary items differently from what was intended. One possibility is that respondents may include experiences from outside of the specified reporting period. For example, for instructional phrases that are apparently more ambiguous, such as *in the last day*, it is easy to imagine that respondents have a different interpretation of the instructions, which could mean either *the day that has just passed* or *yesterday*. It is also notable that none of the instructional phrases explicitly tell the respondents to consider *all* the experiences within the specified reporting period when providing responses. Therefore, it is possible that respondents recall their experience from only a particular period of the specified reporting period (eg, just the morning) and not from the entire reporting period. Both these instances are problematic for interpreting diary data because responses might not be about the periods that the investigators aim to investigate. A concerning implication is that these factors could introduce errors in analyses that examine the relationship between diary data and data collected from other sources (eg, blood pressure, accelerometers, or phone interview data). Therefore, the primary goal of this study was to explore the effectiveness of the instructional phrases at directing respondents to the intended reporting period. Specifically, we examined which time frames participants had in mind when completing daily recall items with different instructional phrases. In addition, as the effectiveness of these items could also be undermined if the instructional phrases are less straightforward or cognitively challenging for participants, we explored whether some instructional phrases were more easily recognized and correctly remembered than others. Finally, it is possible that longer recall periods are more susceptible to the influence of cognitive heuristics, such as the peak-end rule [20], which predicts higher levels of affect, given the enhanced salience of affective peaks when the heuristic is operative. Therefore, we also explored the possibility that the instructions (and periods used for recall) impacted the levels of recalled moods.

## Methods

### Study Design

The study was an experimental design in which participants were randomized to answer 1 of the 4 versions of a daily diary. Although daily diaries typically involve data collection over multiple days, for the present purposes, data were collected only for a single day. All participants were asked to rate the extent to which they felt 12 affective states: happy, content, calm, enthusiastic, excited, relaxed, distressed, frustrated, tense, bored, discontent, and dissatisfied (presented in a randomized order). The 4 experimental groups differed by the phrase that introduced each item: (1) “Today, I felt...” (2) “Since waking up today, I felt...” (3) “During the last 24 hours, I felt...,” or (4) “In the last day, I felt... .”

### Measures

#### Self-Reported Time Frame of Reference

**Figure 1:**
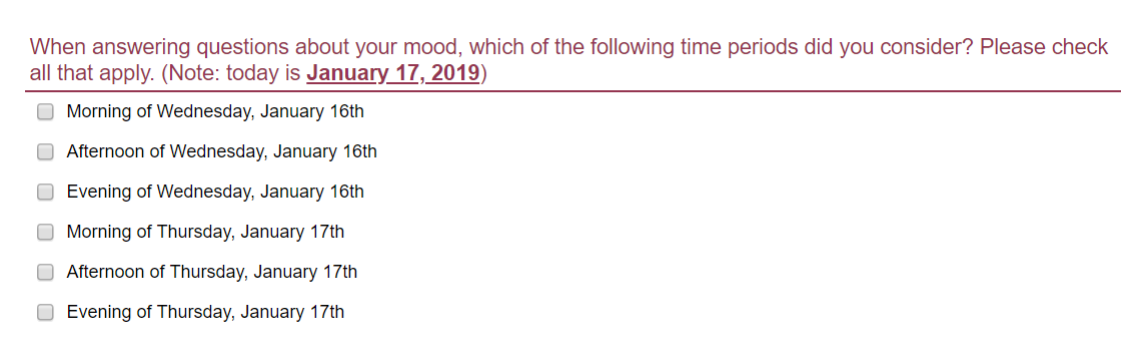
Self-reported time frame of reference for the daily affective state items.

After completing the daily affective states items, participants were asked to select the periods they used when answering these items. Participants were asked, “When answering questions about your mood, which of the following time periods did you consider?” and were presented with 6 time frames: morning today, afternoon today, evening today, morning yesterday, afternoon yesterday, and evening yesterday. The current date was provided at the end of the question and its response options to avoid confusion about the meaning of *today* and *yesterday* (eg, see Figure 1). Participants were asked to select all the time frames that they had considered when rating their affective states.

#### Instruction Recognition Assessment

To examine whether participants recognized and accurately remembered the wording of the time frame presented to them, after selecting the time frames they had considered, they were asked which instructional phrase they had originally received. Participants were provided a list of 5 options that included *today*, *during the last 24 hours*, *since waking up today*, *in the last day*, and *I am not sure*. A response to this question was only considered correct if the participant had selected the option with the instructional phrase to which he or she had been assigned.

### Participants and Procedures

Participants (n=600) in this study were recruited through Amazon Mechanical Turk (MTurk). The study was open to registered MTurk workers (MTurkers) aged at least 18 years who were located in the United States, completed and received approval for at least 500 MTurk tasks (ie, human intelligence tasks), and had a task approval rate of at least 99%. Participants were instructed to complete the EOD survey only between 6 PM and midnight (12 AM). As this study aimed at obtaining participants’ responses at the end of the day, responses from participants who completed the survey before 6:00 PM were excluded. Furthermore, respondents who provided ratings after midnight could be considering the day on which the survey was made available to them (or the date of survey administration) as *the previous day* or *yesterday*. Responses provided after midnight of the survey administration date were, therefore, also excluded. Participants who accepted the task were directed to a Web-based study survey that first asked about demographic information (eg, age, gender, race, ethnicity, education attainment, annual household income, and marital status), followed by an item for identifying carelessly inattentive responders [21]. For this item, participants were asked to choose the synonym for the word *obvious* from a list of 7 words. Participants who did not answer this attention check question correctly were excluded from the analysis. Participants who completed this survey were compensated with US $0.50 (50 cents) through MTurk. The University of Southern California institutional review board approved all the study procedures.

### Statistical Analyses

To evaluate the effectiveness of the instructional phrases for inducing participants to use the expected periods, we classified each time frame response as either acceptable or unacceptable. The criteria for classifying survey respondents are shown in Table 1. Participants’ responses were considered acceptable when they indicated recalling from time frames that are within the intended recall period. Responses were classified as unacceptable only when they were clearly not within the intended time frame. Definitions for *unacceptable* responses differ by the instructional phrases. For *today* and *since waking up today* conditions, participants’ responses were considered unacceptable if they reported drawing reference from any part of yesterday. For the *during the last 24 hours* condition, participants’ responses were considered unacceptable if they reported drawing reference from time frames that (1) included time frames that entailed more than 24 hours or (2) only included a time frame mainly from yesterday. One exception for the second rule was if the participants had selected only yesterday evening because yesterday evening could have been within 24 hours of when the participants started the survey if they started the survey at, or shortly after, 6 PM. The definitions for unacceptable responses for the *in the last day* condition were ambiguous, given that in the last day can be interpreted as *today (ie, in the day that has just passed)*, *yesterday* *(ie, in the previous day)*, or *during the past 24 hours (ie, the notion of day interpreted as 24 hours)*. Owing to the variety of interpretations for this instructional phrase, we felt we could not define acceptable and unacceptable responses. Instead, we present the responses provided by respondents who were in the *in the last day* survey condition descriptively.

**Table 1:** Definitions for acceptable and unacceptable responses for each survey condition.

Survey condition	Acceptable responses	Unacceptable responses
Today and since waking up today	Today morning, afternoon, and evening Today morning and afternoon Today afternoon and evening Today morning only Today afternoon only Today evening only	Yesterday morning only Yesterday afternoon only Yesterday evening only Any combination of time frames within yesterday Any combination of time frames that contains both today and yesterday
During the last 24 hours	Today morning, afternoon, and evening Yesterday evening and today morning, afternoon, and evening Yesterday evening and today morning and afternoon Yesterday afternoon and evening and today morning and afternoon Today evening only Today afternoon only Today morning only Yesterday evening only A combination of 2 time frames within today	If the time frame selected were more than 24 hours If the time frame selected began and ended more than 24 hours away from today evening or afternoon

Chi-square tests were conducted to examine whether the proportion of responses that were considered acceptable (vs unacceptable) differed among the *today*, *since waking up today*, and *during the last 24 hours* conditions or whether participants remembered the assigned instructional phrase correctly. Chi-square tests were also used to determine if there were group differences (over all 4 conditions) in the proportion of individuals correctly remembering the instructional phrase.

Although the primary purpose of the study was to examine self-reported periods evoked by different instructions, we also examined the possibility that the instructions (and the periods used in recall) impacted the reported affect levels. Multivariate analysis of variance (MANOVA) tests were conducted to explore group differences in the average level of mood ratings and whether mood ratings differed among participants who (1) reported recalling only from the day of survey administration or only from the previous day, (2) reported recalling from periods that were immediately before the survey or in some temporal distance from the time of the survey, and (3) reported recalling their mood over shorter versus longer periods. All statistical analyses were conducted using SAS version 9.4 and STATA version 16.

## Results

### Participant Demographics

In total, 600 MTurkers completed the survey. Of these, 13 respondents were excluded because they started the survey after midnight, 2 were excluded because they did not select any time frame for their daily affective states, and 8 were excluded because they did not answer the attention check question correctly. The analytic sample included the remaining 577 adults aged 18 to 75 years (mean 37.57 years, SD 11.43). Approximately half of the sample was male (286/577, 49.6%), 47.7% (275/577) were married, and 90.6% (523/577) had at least some college education (Table 2). Demographic characteristics did not differ across survey conditions or between those who were excluded and those who were included in the analytic sample.

**Table 2:** Demographic characteristics.

Demographic information			Full sample (N=577)		Today (n=146)		Since waking up today (n=145)		In the last day (n=141)		During the last 24 hours (n=145)	
Age (years), mean (SD; range)			37.57 (11.43; 19-75)		38.20 (11.96; 20-66)		37.52 (11.46; 19-75)		37.56 (11.59; 20-71)		37.02 (10.78; 22-71)	
**Gender, n (%)**												
	Male	286 (49.6)		66 (45.2)		76 (52.4)		71 (50.4)		73 (50.3)		
	Female	289 (50.1)		79 (54.1)		69 (47.6)		6971 (48.9)		72 (49.3)		
	Missing	2 (0.4)		1 (0.7)		0 (0.0)		1 (0.7)		0 (0.0)		
**Education, n (%)**												
	High school or less	54 (9.4)		10 (6.9)		12 (8.3)		12 (8.5)		20 (13.5)		
	Some college	142 (24.6)		37 (25.3)		40 (27.6)		30 (21.3)		35 (23.5)		
	Technical school or college degree	299 (51.8)		78 (53.4)		76 (51.0)		78 (55.3)		69 (46.0)		
	Postgraduate degree	82 (14.2)		21 (14.4)		19 (13.1)		21 (14.9)		21 (13.9)		
**Income (US $), n (%)**												
	<20,000	62 (10.8)		12 (8.2)		19 (13.1)		17 (12.1)		14 (9.2)		
	20,000-49,999	203 (35.2)		47 (32.2)		52 (35.9)		45 (31.9)		59 (38.6)		
	50,000-99,999	222 (38.5)		63 (43.2)		54 (37.2)		54 (38.3)		51 (33.1)		
	100,000-150,000	64 (11.1)		16 (11.0)		15 (10.3)		18 (12.8)		15 (9.7)		
	>150,000	26 (4.5)		8 (5.5)		5 (3. 5)		7 (5.0)		6 (3.9)		
**Marital status, n (%)**												
	Married	275 (47.0)		70 (48.0)		70 (48.3)		61 (43.3		69 (44.0		
	Never married	251 (42.9)		63 (43.2)		56 (38.6)		64 (45.4)		66 (41.8)		
	Separated	6 (1.0)		2 (1.4)		1 (0.7)		1 (0.7)		8 (5.0)		
	Divorced	45 (7.7)		6 (4.1)		16 (11.0)		14 (9.9)		2 (1.3)		
	Widowed	8 (1.4)		5 (3.4)		2 (1.4)		1 (0.7)		0 (0.0)		

### Proportion of Participants With Unacceptable Recall Time Frames by Survey Condition

The proportion of respondents who provided recall time frames that were considered *unacceptable* from the *today* (n=146), *since waking up today* (n=145), and *during the last 24 hours* (n=145) conditions were 3.4%, 6.2%, and 31.0%, respectively (Multimedia Appendix 1). The proportion of unacceptable recall time frames differed across the 3 survey conditions (χ^2^_2,436_=57.4; *P*<.001). It was significantly higher in the *during the last 24 hours* (31.0%) condition compared with the *today* (3.4%; χ^2^_1,291_=39.0; *P*<.001) and the *since waking up today* (6.2%; χ^2^_1,290_=29.5; *P*<.001) conditions, whereas it did not differ between the *today* and *since waking up today* conditions (χ^2^_1,291_=1.23; *P*=.27). The proportion of respondents who provided an unacceptable recall time frame was not related to whether the respondents correctly recalled their assigned instructional phrase (χ^2^_1,577_=0.75; *P*=.39).

Respondents in the *in the last day* (n=141) condition reported using the following time frames: just today (49/141, 34.8%), just yesterday (33/141, 23.4%), and a combination of today and yesterday (59/141, 41.8%). The distribution of time frames used by participants in this condition is presented in Multimedia Appendix 2.

### Proportion of Participants Who Correctly Recalled Instructional Phrases by Survey Condition

The proportion of participants who correctly recalled the instructional phrase that they received differed significantly across the 4 conditions (χ^2^_3,577_=145.3; *P*<.001). The proportion was significantly higher in the *today* (91.10%) condition compared with the *during the last 24 hours* (63.5%; χ^2^_1,291_=31.7; *P*<.001) and the *in the last day* (36.9%; χ^2^_1,287_=92.0; *P*<.001) conditions. The proportion was also significantly higher in the *since waking up today* (92.4%) condition compared with the during *the last 24 hours* (χ^2^_1,290_=35.4; *P*<.001) and the *in the last day* (χ^2^_1,286_=97.0; *P*<.001) conditions. The proportions were not different between the *today* and the *since waking up today* conditions (χ^2^_1,291_=0.1670; *P*=.68).

### Impact of Instructions on Levels of Affective States

MANOVA results with instruction group (4 levels) as the independent variable and the 12 affective ratings as dependent variables indicated an overall effect of instructional phrases on self-reported levels of affective states (Wilks lambda, *F*_3,573_=2.14; *P*<.001). Post hoc analyses showed group differences for excited (*F*_3, 573_=4.37; *P*<.005), frustrated (*F*_3,573_=2.39; *P*=.07), content (*F*_3,573_=3.29; *P*=.02), happy (*F*_3,573_=4.09; *P*=.007), and enthusiastic (*F*_3,573_=3.69; *P*=.012). Descriptive information for affective ratings by instruction group is presented in Table 3.

**Table 3:** Descriptive statistics of affective state items by survey condition.

Affective state items	Today (n=146), mean (SD)	Since waking up today (n=145), mean (SD)	In the last day (n=141), mean (SD)	During the last 24 hours (n=145), mean (SD)
Positive affect	4.10 (1.48)	4.25 (1.38)	4.56 (1.44)	4.33 (1.39)
Excited	3.16 (1.69)	3.33 (1.76)	3.84 (1.87)	3.68 (1.79)
Enthusiastic	3.47 (1.92)	3.99 (1.67)	4.12 (1.81)	3.95 (1.69)
Happy	4.34 (1.72)	4.54 (1.56)	4.93 (1.45)	4.78 (1.46)
Calm	4.73 (1.59)	4.62 (1.69)	4.89 (1.64)	4.71 (1.59)
Relaxed	4.48 (1.84)	4.43 (1.65)	4.60 (1.69)	4.37 (1.71)
Content	4.42 (1.93)	4.59 (1.57)	5.00 (1.59)	4.48 (1.72)
Negative affect	2.86 (1.49)	2.72 (1.48)	2.81 (1.44)	3.05 (1.51)
Frustrated	3.07 (1.81)	2.94 (1.88)	2.95 (1.71)	3.44 (1.91)
Distressed	2.73 (1.75)	2.57 (1.70)	2.77 (1.76)	2.84 (1.84)
Tense	3.03 (1.91)	2.88 (1.76)	2.90 (1.73)	3.30 (1.86)
Dissatisfied	3.00 (1.92)	2.73 (1.81)	2.81 (1.80)	2.94 (1.64)
Bored	2.48 (1.63)	2.49 (1.68)	2.76 (1.74)	2.89 (1.85)
Discontent	2.88 (1.89)	2.69 (1.79)	2.66 (1.70)	2.90 (1.75)

Additional exploratory analyses attempted to determine how the endorsement of specific periods was associated with affective states regardless of the experimental condition to which individuals were assigned. MANOVA results indicated no significant difference in affective ratings between the group that reported recalling from yesterday (n=53) and the group that reported recalling today (n=385). Next, we created another variable representing the most distal time point that participants reported considering relative to the time the assessment was completed, ie, for some individuals, yesterday morning was the most distal period, whereas for others, yesterday afternoon was the most distal, and so on. The Ns for the 6 groups that were formed this way were 90, 38, 64, 300, 32, and 53, and the MANOVA of group differences in affect levels was significant (Wilks lambda, *F*_5,571_=1.41; *P*=.02). Post hoc tests showed significant effects only for excited and frustrated states. The pattern for the excited state was difficult to interpret (with the highest scores in groups that considered periods starting at the most distal and most proximal of all periods), whereas for the frustrated state, the highest score was found in the group that considered only the most distal period. Thus, there was not a consistent picture that emerged from these analyses.

Finally, we examined the number of periods endorsed by participants to address the speculation that more periods would afford a higher chance of experiencing a peak affective state than having a shorter reporting period. The MANOVA was not significant.

## Discussion

### Principal Findings

Data collected using daily diary methods can provide insights into participants’ daily lives. Although the utility of the method has been documented extensively across many disciplines, how the instructional phrases are interpreted by survey respondents has not been examined. This study found that periods respondents reported using for answering diary questions are considerably different across 4 common instructional phrases in EOD diary studies. These findings have implications for designing daily diary studies because recall data from unintended recall time frames could threaten the validity of the data and could yield misleading results when analyzing relationships among day-level data.

We found that most respondents of the *today* and *since waking up today* conditions reported using time frames that we believe study investigators intended to capture. These instructional phrases are, in our view, effective in directing participants to recall from the correct time frames, possibly because they are cognitively simple to process. Results from the instruction recall assessment also support this notion, as the vast majority of participants from these groups (91.1% in the *today* group and 92.4% in the *since waking up today* group) correctly recognized their instructional phrases. However, it is important to note that, although these instructions may be easy for respondents to process, some still provided less than optimal responses. We found that a small to moderate proportion of respondents in both instructional phrase groups (*today* group: 19.9% [29/146] and *since waking up today* group: 30.3% [44/145]) reported recalling from only short segments of time, as opposed to longer periods within the day. Although these participants were using periods that were within the boundary of *today*, not using all the intended periods could introduce bias to the collected data [22].

With regard to the *during the last 24 hours* instructional phrase, 69.0% of the respondents reported using periods that resembled the 24 hours before the survey administration (eg, from the morning of today to the evening of today, from the afternoon of yesterday to the afternoon of today, etc). Considering that 63.8% of respondents in this group correctly recognized their assigned daily item instructional phrase, it is possible that *during the last 24 hours* is cognitively challenging for respondents to process, at least compared with the 2 more straightforward instructional phrases examined in this study. Thus, this phrase is potentially less effective for use in diary studies.

Finally, we found that participants in the *in the last day* group reported using a large variety of time frames. Our results indicated that a large portion of respondents (41.8%) in this group interpreted this instructional phrase as a combination of both today and yesterday, whereas others in the same group interpreted the phrase *in the last day* as *today* (34.8%) or *yesterday* (23.4%). The variety of recall periods reported in this group raises concerns about the effectiveness of this instructional phrase in directing participants to recall their experiences in the way diary researchers intended. The likely reason for the variety of recall period patterns reported here is that the instructional phrase *in the last day* was ambiguous, as only 35.1% of respondents in this group correctly recognized their survey instructions. The heterogeneity of recall time frames reported here highlights the need for a better understanding of how survey respondents comprehend and respond to this instructional phrase.

It is also important to note that the average levels of some affects significantly differed by time frame instructions. Previous studies have documented that retrospective self-reports of mood depend on the length of the reporting period (such that longer reporting periods are often associated with higher positive and negative affect reports) [22-24]. Although those studies compared instructions for reporting periods that varied considerably in length (from moments to weeks and months and years), the present results suggest that even differences in instructions for ostensibly very similar reporting periods (ie, a day) can affect the levels of self-reported experiences. This may confound the comparability of results of daily diary studies that use different instructions in the diary because participants using different instructions may actually be reporting about different periods.

Our exploratory analyses to examine whether the endorsement of specific periods was associated with affect levels yielded mixed results, with no consistent evidence that the length or proximity of the periods that participants considered systematically impacted the ratings. However, these exploratory analyses were observational in nature (ie, they were conducted, regardless of the experimental condition to which individuals were assigned) and required replication using larger samples and possibly using experimental manipulation.

We believe that these results suggest several recommendations for future daily diary studies. If a researcher would like to capture experience about the current day, then the results clearly show that *today* and *since waking up today* instructional phrases are effective in directing participants to the intended periods of the day. Nevertheless, there is room for improvement even in these instructions, given the modest error rates we found. This suggests that a more thorough set of instructions is in order, perhaps with the inclusion of examples to make the task very clear. We suspect that there will always be some participants who will not or cannot follow instructions, but we also believe that better instructions, such as encouraging participants to recall their experience for today as a whole, rather than only for parts of today, could be helpful. Our recommendation for the *during the last 24 hours* and *in the last day* is also straightforward: clearly specify the date and time frame for participants, as these instructional phrases produce a wide variety of recall periods. The instruction *during the last 24 hours* does not appear to be tapping what we believe researchers intend. Perhaps, this phrasing could be effective if participants were provided examples of which periods should be considered, but this remains to be seen. If the intent for using the *in the last day* instructional phrase is to get at the current day, then we recommend using one of the first 2 instructional phrases instead. If the *in the last day* instruction is intended for time frames other than *the day of survey administration*, then it may be in the researchers’ best interest to clearly specify the intended date and time frame of reference. For all the instructional phrases, we recommend instructing respondents to consider the entire period of time and not just segments of the day.

### Limitations

Although the results of this study offer insights into the recall time frame of 4 commonly used instructional phrases, there are limitations to the results. One limitation is the fact that this study was conducted with MTurkers. There is a growing body of literature documenting that MTurkers are not comparable with the general population in many ways. The current literature suggests that MTurkers are different from the general US population in some demographic characteristics (eg, are younger, received more years of education, have lower income, and are less ethnically diverse [25]) and in psychological characteristics (eg, more cognitive symptoms [26], more likely to be depressed, anxious, or socially isolated [26,27], and report lower in subjective well-being [28]). However, other evidence has also suggested that MTurkers are more attentive to task instructions [29]. These documented differences suggest the need for future studies to replicate the findings of this study using more diverse and representative samples. In addition, participants in this study were asked to complete the diary items only at a single time point, whereas daily diaries are completed multiple times across consecutive days in most diary studies (for exceptions, refer to the studies by Stone et al [30] and Stone et al [31]). We do not think this invalidates our results, but it is possible that the interpretation of instructional phrases for daily diary items changes after repeated administration. Finally, our results assume that participants can veridically report on the periods they considered when answering diary questions. Some may question that supposition, and we are hard-pressed to provide evidence to the contrary. Nevertheless, we believe the methods are likely to produce informative data.

### Conclusions

In summary, this study showed that EOD diary instructional phrases may not always be interpreted by survey respondents in the way that the investigators intended. Among the 4 commonly used instructional phrases, the *today* and the *since waking up today* phrases were the most effective in capturing respondents’ experience on the day of inquiry. We recommend that the phrases *in the last 24 hours* and *in the last day* be used with much caution—if at all—given the lack of consistent periods being selected by participants.

## Appendix

Multimedia Appendix 1 Descriptive statistics of participants’ self-report recall time frame by survey conditions.

Multimedia Appendix 2 Descriptive statistics of participants’ self-report recall time frame in the in the last day survey condition.

## Abbreviations

EOD: end-of-day
MANOVA: multivariate analysis of variance
