# Supplementary material for: Evaluating the Effect of Daily Diary Instructional Phrases on Respondents’ Recall Time Frames: Survey Experiment
Source: J Med Internet Res. 2020 Feb 21;22(2):e16105. doi: 10.2196/16105 (PMC7060498; doi:10.2196/16105)
Supplement: Multimedia Appendix 1 [file jmir_v22i2e16105_app1.docx]

| Survey condition | Response category | Yesterday | | | Today | | | N(%) | Start time (hh:mm) | |
| --- | --- | --- | --- | --- | --- | --- | --- | --- | --- | --- |
|  |  | Morning | Afternoon | Evening | Morning | Afternoon | Evening |  | Count | Mean (SD) |
|  | | | | | | | | | | |
| *Today* (N=146) | Acceptable |  |  |  |  |  |  | 141 (96.6%) | 97 | 21:41 (1:16) |
|  |  |  |  |  |  |  |  |  | 12 | 20:47 (1:24) |
|  |  |  |  |  |  |  |  |  | 3 | 21:37 (1:07) |
|  |  |  |  |  |  |  |  |  | 14 | 21:16 (1:27) |
|  |  |  |  |  |  |  |  |  | 8 | 21:52 (1:32) |
|  |  |  |  |  |  |  |  |  | 7 | 21:52 (1:20) |
|  | Unacceptable |  |  |  |  |  |  | 5 (3.4%) | 1 | 21:45 (—^a^) |
|  |  |  |  |  |  |  |  |  | 3 | 22:18 (0:56) |
|  |  |  |  |  |  |  |  |  | 1 | 22:17 (—) |
| *Since waking up today*” (N=145) | Acceptable |  |  |  |  |  |  | 136 (93.7%) | 76 | 21:47 (1:12) |
|  |  |  |  |  |  |  |  |  | 3 | 22:31 (1:35) |
|  |  |  |  |  |  |  |  |  | 13 | 21:21 (1:26) |
|  |  |  |  |  |  |  |  |  | 13 | 21:51 (1:35) |
|  |  |  |  |  |  |  |  |  | 7 | 21:56 (1:11) |
|  |  |  |  |  |  |  |  |  | 24 | 22:13 (1:17) |
|  | Unacceptable |  |  |  |  |  |  | 9 (6.2%) | 1 | 21:12 (—) |
|  |  |  |  |  |  |  |  |  | 1 | 22:13 (—) |
|  |  |  |  |  |  |  |  |  | 1 | 23:36 (—) |
|  |  |  |  |  |  |  |  |  | 4 | 21:31 (0:56) |
|  |  |  |  |  |  |  |  |  | 1 | 20:13 (—) |
|  |  |  |  |  |  |  |  |  | 1 | 18:13 (—) |
| *During the last 24 hours* (N=145) | Acceptable |  |  |  |  |  |  | 100 (69.0%) | 31 | 21:57 (1:30) |
|  |  |  |  |  |  |  |  |  | 32 | 21:18 (1:30) |
|  |  |  |  |  |  |  |  |  | 4 | 21:41 (2:15) |
|  |  |  |  |  |  |  |  |  | 2 | 22:03 (2:19) |
|  |  |  |  |  |  |  |  |  | 2 | 20:15 (2:25) |
|  |  |  |  |  |  |  |  |  | 15 | 21:38 (1:19) |
|  |  |  |  |  |  |  |  |  | 5 | 20:54 (1:36) |
|  |  |  |  |  |  |  |  |  | 6 | 21:26 (1:01) |
|  |  |  |  |  |  |  |  |  | 3 | 22:18 (0:27) |
|  | Unacceptable |  |  |  |  |  |  | 45 (31.0%) | 6 | 21:13 (2:01) |
|  |  |  |  |  |  |  |  |  | 3 | 20:57 (0:34) |
|  |  |  |  |  |  |  |  |  | 3 | 21:28 (2:02) |
|  |  |  |  |  |  |  |  |  | 1 | 23:28 (—) |
|  |  |  |  |  |  |  |  |  | 21 | 22:27 (1:07) |
|  |  |  |  |  |  |  |  |  | 6 | 22:20 (1:06) |
|  |  |  |  |  |  |  |  |  | 3 | 22:05 (0:46) |
|  |  |  |  |  |  |  |  |  | 1 | 23:44 (—) |
|  |  |  |  |  |  |  |  |  | 1 | 20:40 (—) |

^a^—: not applicable.
