# Supplementary material for: Evaluating the Effect of Daily Diary Instructional Phrases on Respondents’ Recall Time Frames: Survey Experiment
Source: J Med Internet Res. 2020 Feb 21;22(2):e16105. doi: 10.2196/16105 (PMC7060498; doi:10.2196/16105)
Supplement: Multimedia Appendix 2 [file jmir_v22i2e16105_app2.docx]

|  |  |  |  |  |  |  |  |  |
| --- | --- | --- | --- | --- | --- | --- | --- | --- |
| Survey condition | Yesterday | | | Today | | | Start time (hh:mm) | |
|  | Morning | Afternoon | Evening | Morning | Afternoon | Evening | Count | Mean (SD) |
|  | | | | | | | | |
| ***In the last day*** | | | | | | | | |
|  |  |  |  |  |  |  | 11 | 22:01 (1:43) |
|  |  |  |  |  |  |  | 1 | 20:23 (—^a^) |
|  |  |  |  |  |  |  | 26 | 21:50 (1:21) |
|  |  |  |  |  |  |  | 3 | 20:58 (1:50) |
|  |  |  |  |  |  |  | 3 | 21:15 (0:04) |
|  |  |  |  |  |  |  | 5 | 21:38 (1:46) |
|  |  |  |  |  |  |  | 3 | 22:40 (0:37) |
|  |  |  |  |  |  |  | 1 | 22:42 (—) |
|  |  |  |  |  |  |  | 19 | 21:49 (1:39) |
|  |  |  |  |  |  |  | 6 | 21:43 (1:11) |
|  |  |  |  |  |  |  | 1 | 22:08 (—) |
|  |  |  |  |  |  |  | 3 | 20:44 (2:49) |
|  |  |  |  |  |  |  | 17 | 21:17 (1:23) |
|  |  |  |  |  |  |  | 7 | 21:59 (1:18) |
|  |  |  |  |  |  |  | 19 | 21:49 (1:06) |
|  |  |  |  |  |  |  | 3 | 20:37 (2:07) |
|  |  |  |  |  |  |  | 2 | 20:45 (2:10) |
|  |  |  |  |  |  |  | 4 | 21:32 (1:36) |
|  |  |  |  |  |  |  | 1 | 23:47 (—) |
|  |  |  |  |  |  |  | 3 | 21:38 (2:09) |
|  |  |  |  |  |  |  | 1 | 22:19 (—) |
|  |  |  |  |  |  |  | 1 | 21:55 (—) |
|  |  |  |  |  |  |  | 1 | 22:34 (—) |

^a^—: not applicable.
